# Cell recovery by reversal of ferroptosis

**DOI:** 10.1242/bio.043182

**Published:** 2019-06-15

**Authors:** Ho Man Tang, Ho Lam Tang

**Affiliations:** 1 Institute for Basic Biomedical Sciences, Johns Hopkins University School of Medicine, Baltimore, MD 21205, USA; 2 School of Life Sciences, Chinese University of Hong Kong, Shatin NT, Hong Kong, China; 3 Department of Neurosurgery, Johns Hopkins University School of Medicine, Baltimore, MD 21205, USA

**Keywords:** Anastasis, Ferrostatin-1, Glutamate, Glutathione, Reversal of apoptosis, Reversal of ferroptosis

## Abstract

The classical view of cell death has long assumed that, once initiated, the dying process is irreversible. However, recent studies reveal that recovery of dying cells can actually occur, even after initiation of a cell suicide process called apoptosis. This discovery raised fundamental key questions about which forms of the cell death process could be reversible and how reversal is mediated. Here, we uncover an unanticipated reversibility of ferroptotic cell death process. Unlike apoptosis reversal, removal of ferroptosis inducers, such as erastin and glutamate, is insufficient to allow ferroptotic dying cells to escape the cell death process. However, by removing the cell death inducer and providing the reduced form of glutathione or the radical-trapping antioxidant ferrostatin-1, ferroptotic dying cells can be rescued and promoted to recover. Interestingly, although ferroptotic inhibitors such as aminooxyacetic acid, deferoxamine, dopamine and vitamin C can prevent initiation of ferroptosis, added alone they are unable to reverse the initiated ferroptosis, suggesting regulatory distinctions between preventing and reversing ferroptosis. Together, these results reveal the first evidence that ferroptosis is reversible and suggest strategies to enhance its reversibility, thereby providing a useful model for studying the physiological, pathological and therapeutic potentials of this cell recovery process.

## Abstract (precis)

**Summary:** Programmed cell death is generally assumed to be irreversible. Interestingly, we discovered reversible apoptosis. Here, we further demonstrate reversible ferroptosis, thereby adding a new example of reversible programmed cell death.

## INTRODUCTION

Programmed cell death plays essential roles in embryonic development and normal homeostasis by eliminating unwanted, injured or dangerous cells from the body (Jacobson et al., 1997; Fuchs and Steller, 2011). Targeting regulators of cell death is also a therapeutic strategy against intractable diseases such as cancer, heart failure, and degeneration (Johnstone et al., 2002; Hotchkiss et al., 2009; Morgan and Harris, 2015; Menasché, 2018). It is widely believed that initiated programmed cell death, such as apoptosis, is irreversible (Alberts et al., 2002, 2014; Holland and Cleveland, 2012). Challenging this general assumption, we discovered that the apoptotic process is reversible even at late stages of the cell death process, both *in vitro* and *in vivo* (Tang et al., 2009, 2012, 2015a,b, 2017; Tang and Tang, 2018). We coined a term, ‘anastasis’ (Greek for ‘rising to life’), to describe the recovery of dying cells after they reach the brink of death, using reversal of apoptosis as the first example (Tang et al., 2012; Tang and Tang, 2018). Removal of the cell death stimuli is sufficient to allow apoptotic dying cells to recover, indicating that this is an intrinsic recovery phenomenon (Tang et al., 2009, 2012, 2015a,b, 2017; Wang et al., 2014; Ding et al., 2016; Sun et al., 2017; Xu et al., 2018; Tang and Tang, 2018). Since there are multiple forms of programmed cell death (Galluzzi et al., 2018), the discovery of anastasis leads to an intriguing and fundamental question: which forms of programmed cell death are reversible?

Ferroptosis is a non-apoptotic, iron-dependent form of programmed cell death (Dixon et al., 2012; Galluzzi et al., 2018). This form of cell death is defined by a requirement for iron and an accumulation of cellular reactive oxygen species (ROS) for this cell death execution (Dixon et al., 2012; Stockwell et al., 2017; Galluzzi et al., 2018). Ferroptosis can be triggered by natural stimuli such as the neurotransmitter glutamate and by synthetic agents such as the small molecule erastin (Dixon et al., 2012; Stockwell et al., 2017), and can be efficiently blocked by iron chelators and antioxidants (Dixon et al., 2012; Stockwell et al., 2017; Galluzzi et al., 2018). Ferroptotic cells do not display classic morphological and biochemical hallmarks of apoptosis such as mitochondrial fragmentation, nuclear condensation, cell shrinkage, plasma membrane blebbing and caspase-3 activation (Dixon et al., 2012; Galluzzi et al., 2018). By contrast, morphological characteristics of ferroptosis include cell rounding followed by plasma membrane rupture at the end stage of this cell death process (Dixon et al., 2012; Linkermann et al., 2014; Galluzzi et al., 2018). Lipid peroxidation also occurs in ferroptotic cells due to the accumulation of ROS (Feng and Stockwell, 2018).

Is ferroptosis reversible? Here, we find that, different from reversal of apoptosis, removing cell death stimuli from the culture medium of rounded-up ferroptotic dying cells is not sufficient to promote reversal of ferroptosis. Inhibitors of ferroptosis, such as the small molecule transaminase inhibitor aminooxyacetic acid (AOA), the iron chelator deferoxamine (DFO), the neurotransmitter dopamine, and the antioxidant vitamin C, block cell death only when they are added together with ferroptosis stimulus, but they fail to rescue dying cells if added after ferroptosis has been induced. Interestingly, however, two other compounds, the reduced glutathione (GSH) and the radical-trapping antioxidant ferrostatin-1 (Fer-1), not only suppress the initiation of ferroptosis (as above) but can also promote its reversal when added after removing the ferroptotic stimulus. Therefore, the present study provides a new example of anastasis by revealing ferroptosis reversal. Our findings also suggest strategies to mediate the reversibility of ferroptosis, with therapeutic implications for tissue injuries and cancers in which involvements of ferroptosis are beginning to emerge.

## RESULTS

### Removal of ferroptosis inducer is not sufficient to trigger reversal of ferroptosis

**Fig. 1.**
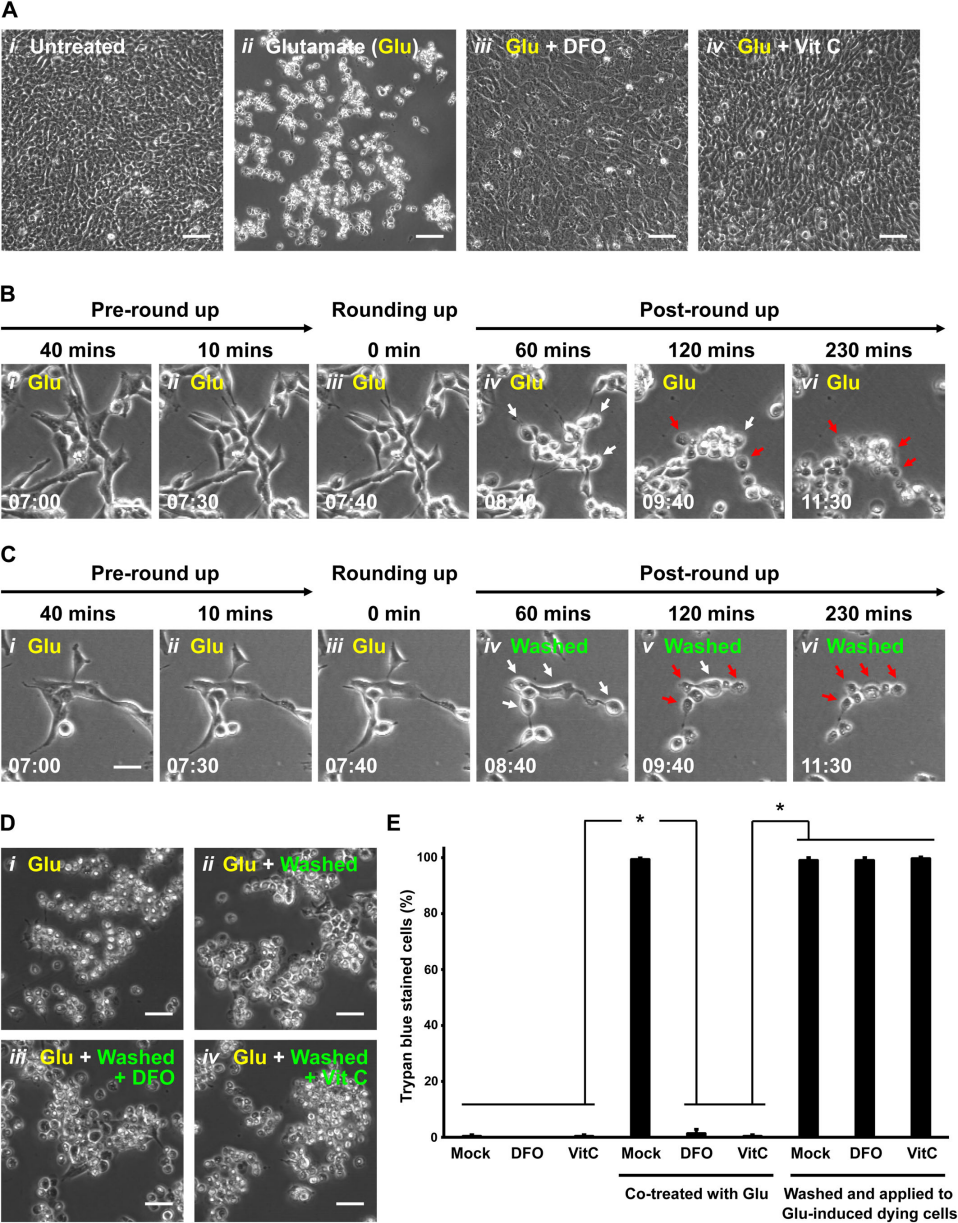
**Demise of ferroptotic cells after removing cell death stimulus.** (A) HT-22 cells were cultured in (*i*) cell medium, (*ii*) medium containing 10 mM glutamate (Glu) for 24 h, (*iii*) medium containing both 10 mM Glu and 100 µM deferoxamine (DFO) for 24 h, and (*iv*) medium containing both 10 mM Glu and 0.5 mM vitamin C (Vit C) for 24 h. Cell morphology was observed and recorded by phase contrast microscopy. Scale bars: 100 µm. (B) Time-lapse live-cell phase contrast microscopy of (*i*,*ii*) pre-rounding, (*iii*) rounding and (*iv–vi*) post-rounding HT-22 cells exposed to 10 mM Glu. Same group of cells shown over time (h:min). Scale bar: 20 μm. White arrows indicate a few examples of rounded-up ferroptotic dying cells. Red arrows indicate collapsed ferroptotic cells with plasma membrane rupture. (C) Time-lapse live-cell phase contrast microscopy of HT-22 cells during 10 mM Glu induction and after being washed and incubated with fresh medium. Shown here is a group of cells in the culture after induction with Glu at 40 min (*i*) and 10 min (*ii*) prior to showing signs of rounding, at the time rounding began with Glu still in the medium (*iii*) and then at 60, 120 and 230 min after washing and continuing incubation in fresh medium containing no Glu (panels *iv–vi*). Same group of cells shown over time (h:min). Scale bar: 20 μm. White arrows indicate rounded-up ferroptotic dying cells. Red arrows indicate collapsed ferroptotic cells with plasma membrane rupture. (D) Phase contrast microscopy of HT-22 cells treated with 10 mM Glu for 24 h (*i*), treated with 10 mM Glu for 7.5 h to initiate ferroptosis and then washed and incubated with fresh culture medium for 24 h (*ii*), treated as in *ii* but with 100 µM DFO added to the fresh culture medium (*iii*), treated as *ii* but with 0.5 mM Vit C added to the fresh culture medium (*iv*). Scale bars: 60 μm. (E) Percentage of HT-22 cells that displayed plasma membrane permeability in Trypan Blue exclusion assay, after the following treatments. First set of three show culture medium alone (Mock), medium containing 100 µM DFO or 0.5 mM Vit C for 24 h. Second set of three are as in the first set, but all cultures containing 10 mM Glu. Third set of three are as in the first set, but with all cultures induced with 10 mM Glu for 7.5 h to initiate ferroptosis prior to washing and then incubating the rounded-up ferroptotic cells for 24 h with fresh culture medium alone (Mock), or containing 100 mM DFO or 0.5 mM Vit C. Dead cells with plasma membrane rupture displayed full plasma membrane permeability as determined by the Trypan Blue exclusion assay. Data presented as means±s.d. of three independent experiments. Student's *t*-test: *P<0.001.

It has been demonstrated that ferroptosis can be triggered by natural stimuli such as the neurotransmitter glutamate in the immortalized mouse hippocampal neuronal HT-22 cells (Xie et al., 2016; Stockwell et al., 2017). Therefore, this served as our first study model to examine the reversibility of ferroptosis. To begin, we first verified that the ferroptotic cell death process can be triggered by glutamate using this neuronal cell line. Healthy HT-22 cells displayed a flat spreading morphology adherent to the substrate (Fig. 1A*i*). As expected (Xie et al., 2016; Stockwell et al., 2017), after inducing ferroptosis by adding 10 mM of glutamate for 7 to 7.5 h, the HT-22 cells showed a morphological feature of ferroptosis that these dying cells first started to round up (Fig. 1B*i*,*ii*). These rounding-up cells then proceeded to a spherical morphology (Fig. 1B*iii*,*iv*), followed by plasma membrane rupture in the fully rounded-up cells (Fig. 1A*ii*,B*v*,*vi*, Movie 1, Fig. S1A). These cells did not display hallmarks of apoptosis such as cell shrinkage, plasma membrane blebbing, nuclear condensation and caspase-3 activation (Fig. S2A,B). Also as expected, the initiation of ferroptosis can be blocked by ferroptosis inhibitors, such as DFO and vitamin C, when either were added to the culture medium together with glutamate (Fig. 1A*iii*,*iv*, Fig. S1B). This indicates that glutamate can trigger an iron-dependent cell death process in HT-22 cells that requires accumulations of ROS, the hallmarks of ferroptosis (Dixon et al., 2012; Xie et al., 2016; Stockwell et al., 2017).

Is the ferroptotic cell death process reversible? To address this question, we tested the reversibility of ferroptosis by washing and incubating the glutamate-induced rounded-up ferroptotic HT-22 cells with fresh culture medium prior to plasma membrane rupture (Fig. 1C*i*–*iii*). Cells able to reverse ferroptosis are expected to recover and regain a morphology comparable to untreated cells. However, our time-lapse live-cell microscopy showed that, instead of recovering, the washed ferroptotic cells continued their progression to death, ending in plasma membrane rupture (Fig. 1C*iv*–*vi*, Movie 2, Fig. S1B). This outcome contrasts with the reversal of apoptosis and escape from death (anastasis) observed when stimuli of apoptosis are removed (Tang et al., 2009, 2012, 2015a,b, 2017; Wang et al., 2014; Ding et al., 2016; Sun et al., 2017; Xu et al., 2018; Tang and Tang, 2018). We tested and confirmed that HT-22 cells, like other cells tested (Tang et al., 2009, 2012,b; Tang and Tang, 2018), did show a robust apoptosis reversal after washing away ethanol or staurosporine (Fig. S3), two inducers of apoptosis. These results indicate that simply removing ferroptosis inducer from the culture medium is not sufficient to promote its reversal.

DFO and vitamin C can block the initiation of ferroptosis (Fig. 1A*iii*,*iv*). Can these ferroptosis inhibitors promote reversibility of ferroptosis when they are applied to the ferroptotic dying cells? To answer this question, we replaced the culture medium of glutamate-induced rounded-up ferroptotic dying cells, prior to plasma membrane rupture, with fresh medium containing DFO or vitamin C. Morphological changes in the dying cells consistent with reversal of ferroptosis were not observed (Fig. 1D*i*–*iv*), whether incubation was continued in the presence of glutamate (Fig. 1D*i*), glutamate was removed from the culture medium (Fig. 1D*ii*) or glutamate was removed and either DFO or vitamin C were added to the rounded-up ferroptotic cells (Fig. 1D*iii*,*iv,* Fig. S1B) with their optimal dosages or higher that could inhibit the initiation of ferroptosis (Fig. S4A,B). In all cases, glutamate-treated cells progressed to death as determined by plasma membrane permeability with Trypan Blue dye exclusion assay (Fig. 1E). Considered together with the data in Fig. 1A, this indicates that DFO and vitamin C can inhibit the initiation of ferroptosis (Fig. 1A*iii*,*iv*), but are unable to rescue ferroptotic dying cells (Fig. 1D*iii*,*iv*,E).

### Ferrostatin-1 promotes reversal of glutamate-induced ferroptosis

**Fig. 2.**
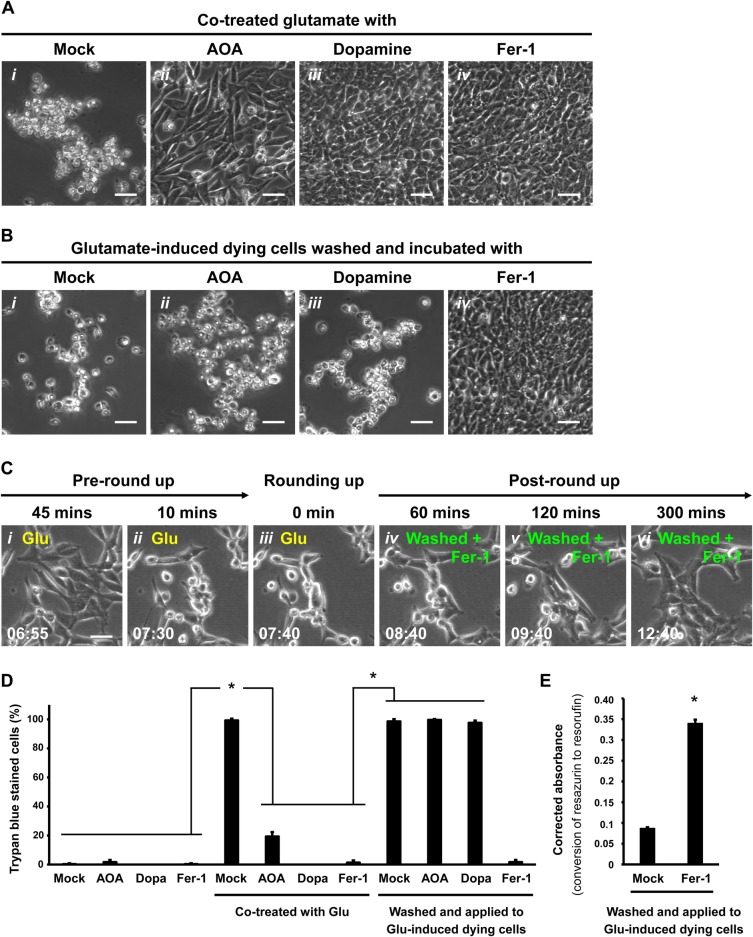
**Reversal of ferroptosis by Fer-1 in glutamate-induced HT-22 dying cells.** (A) Phase contrast images of HT-22 cells cultured in (*i*) medium containing 10 mM Glu for 24 h, (*ii*) medium containing both 10 mM Glu and 2 mM AOA for 24 h, (*iii*) medium containing both 10 mM Glu and 5 µM dopamine for 8 h or (*iv*) medium containing both 10 mM Glu and 10 µM Fer-1 for 24 h. Scale bars: 60 μm. (B) Phase contrast images of HT-22 cells treated with 10 mM Glu for 7.5 h to initiate ferroptosis (time for cell rounding), and then washed and incubated with (*i*) fresh culture medium alone (Mock), (*ii*) fresh medium containing 2 mM AOA, (*iii*) fresh medium containing 5 µM dopamine or (*iv*) fresh medium containing 10 µM Fer-1 for 24 h. Scale bars: 60 μm. (C) Time-lapse live-cell phase contrast microscopy of HT-22 cells showing reversal of ferroptosis by Fer-1. Cells were induced with 10 mM Glu and imaged 45 min and 10 min before showing signs of rounding (*i*,*ii*), the time rounding was first noted (*iii*), and 60, 120 and 300 min after washing to remove Glu and continuing incubation in medium containing 10 µM Fer-1 (*iv*–*vi*). Same group of cells shown over time (h:min). Scale bar: 20 μm. (D) Percentage of HT-22 cells that displayed plasma membrane permeability in Trypan Blue exclusion assay (dead cells), after being treated as follows. First set of four cultures had nothing added to the medium (Mock), or contained 2 mM AOA, 5 µM dopamine (Dopa), or 10 µM Fer-1. The second set of cultures were like the first set, but also contained 10 mM Glu in the culture medium. The third set of cultures were induced for 7.5 h to initiate ferroptosis with 10 mM Glu and then washed and incubated 24 h with medium containing no additive (Mock), or with added 2 mM AOA, 5 µM Dopa or 10 µM Fer-1. Dead cells were scored as those showing full plasma membrane permeability to Trypan Blue in a viability assay. (E) Corrected absorbance for reduction of resazurin to resorufin in HT-22 cells induced with 10 mM glutamate for 7.5 h to initiate ferroptosis, and then washed and incubated 24 h with fresh medium containing either no additive (Mock) or with 10 µM Fer-1. Data presented as means±s.d. of three independent experiments. Student's *t*-test: *P<0.001.

Can arresting different or downstream ferroptotic events rescue ferroptotic dying cells? To answer this question, we tested the effect of several known inhibitors on ferroptotic dying cells. We used AOA that inhibits fatty acid synthesis (Wise et al., 2008), neurotransmitter dopamine that blocks degradation of glutathione peroxidase 4 (GPX4) (Wang et al., 2016), and lipid ROS scavenger Fer-1 (Skouta et al., 2014). All of these inhibitors can block the initiation of ferroptosis in HT-22 cells when added together with glutamate at the beginning of incubation (Fig. 2A*i–iv*). We then tested the inhibitors on rounded-up ferroptotic dying cells immediately after removing glutamate and washing with fresh culture medium, prior to plasma membrane rupture. Although AOA and dopamine were able to suppress the initiation of ferroptosis (Fig. 2A*i–iii*), neither was able to reverse it after the dying cells had displayed the rounded-up morphology (Fig. 2B*i–iii,* Fig. S1B), even with the optimal or higher dosages that were able to suppress initiation of ferroptosis (Fig. S4C,D). In contrast, Fer-1 was able to both inhibit the initiation of ferroptosis (Fig. 2A*iv*) and to reverse its progression to cell death when the glutamate inducer was removed (Fig. 2B*iv,* Fig. S1B). Our time-lapse live-cell microscopy showed that rounded-up ferroptotic cells were able to regain a normal appearance after rinsing away the glutamate and growing in fresh medium containing Fer-1 (Fig. 2C, Movie 3). These rescued cells were not rare escapers, as over 96% of the washed cells cultured with Fer-1 can reverse ferroptosis and survive (Fig. 2D). Proliferation was detected after reversal of ferroptosis, indicating cell recovery (Movie 3). Reversal of ferroptosis by Fer-1 was also supported by our finding that the intracellular reducing environment of recovered cells was restored, indicating them as the metabolically active viable cells. These recovered cells showed chemical reduction of resazurin to resorufin (Erb and Ehlers, 1950) detected by using the resazurin-based PrestoBlue reagent, consistent cell viability (Fig. 2E), whereas ferroptotic cells that had been washed and cultured in medium lacking Fer-1 were not viable as indicated by this assay (Fig. 2E) and also the Trypan Blue assay (Fig. 2D). These results indicate that Fer-1 can promote reversal of glutamate-induced ferroptosis in HT-22 cells.

### Glutathione promotes reversal of glutamate-induced ferroptosis

The reversal of ferroptosis by the radical-trapping antioxidant Fer-1 suggested that restoring the redox environment by other means may also allow cell recovery. Therefore, we tested the efficacy of glutathione as pro-ferroptosis reversal agent. Reduced glutathione (GSH) is an intracellular antioxidant that serves as a cofactor for the selenoenzyme GPX4, which is a GSH-dependent lipid hydroperoxidase required for the clearance of lipid ROS (Ursini et al., 1985). During ferroptosis, the increase of the iron-dependent lipid ROS accumulation exceeds the capability of GSH-dependent GPX4 to convert lipid hydroperoxides to lipid alcohol, leading to defective lipid peroxide repair and lipid peroxidation, the hallmark of ferroptosis that causes cell death (Dixon et al., 2012; Yang and Stockwell, 2016). The collapse of this cellular redox homeostasis can be prevented by adding GSH, which has been shown to block the initiation of ferroptosis (Dixon et al., 2012). As lipid peroxidation is the critical downstream event in the ferroptotic cell death process, we tested the ability of GSH to restore redox homeostasis and rescue ferroptotic dying cells.

**Fig. 3.**
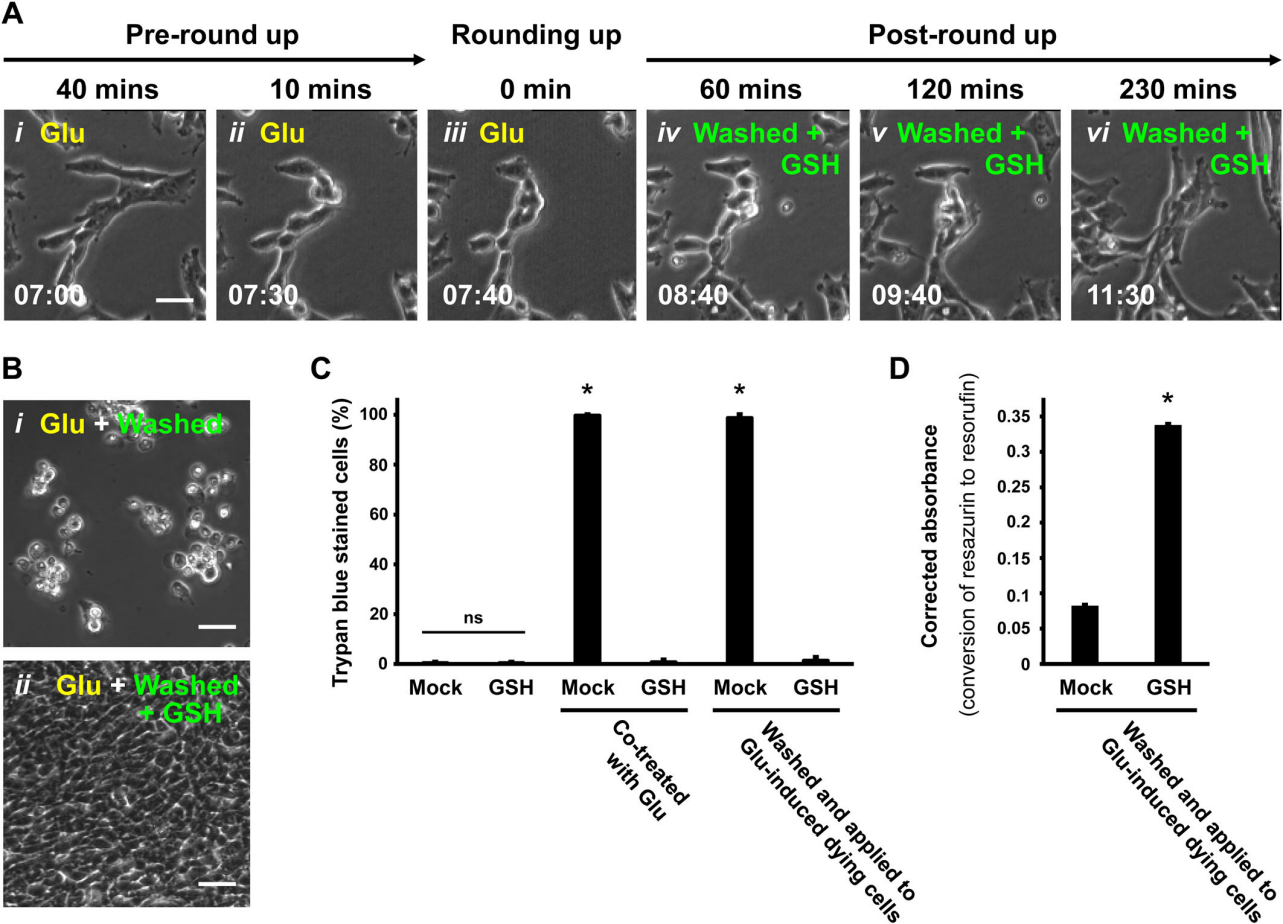
**Reversal of ferroptosis by GSH in glutamate-induced HT-22 dying cells.** (A) Time-lapse live-cell phase contrast microscopy of HT-22 cells showing ferroptosis reversal by GSH. Cells were induced with 10 mM Glu and imaged 40 min and 10 min before showing signs of rounding (*i*,*ii*) and the time rounding was first noted (*iii*), and then 60, 120 and 230 min after washing to remove Glu and continuing incubation in medium containing 1.2 mM GSH (*iv*–*vi*). Same group of cells shown over time (h:min). Scale bar: 20 μm. (B) Phase contrast microscopy of HT-22 cells induced with 10 mM Glu for 7.5 h to trigger ferroptosis, and then washed and incubated for 24 h with either (*i*) fresh culture medium alone or (*ii*) medium containing 1.2 mM GSH. (C) Percentage of HT-22 cells permeable to the vital stain Trypan Blue (dead cells), 24 h after the following treatments. The first set of cultures received no additions (Mock) or 1.2 mM GSH. The second set was treated with 10 mM Glu only (Mock) or with Glu+1.2 mM GSH. The third set was first induced by adding 10 mM Glu to the medium for 7.5 h (time of cell rounding) and then washed and incubated with fresh medium (Mock) or with 1.2 mM GSH. Dead cells were counted as those showing plasma membrane rupture with full permeability to the vital stain Trypan Blue. (D) Corrected absorbance for the reduction of resazurin to resorufin in HT-22 cells induced with 10 mM Glu for 7.5 h to initiate ferroptosis, and then washed and incubated 24 h with fresh medium containing either no additive (Mock) or 1.2 mM GSH. Data presented as means±s.d. of three independent experiments. Student's *t*-test: *P<0.001. ns, not significant.

To do this, we triggered ferroptosis by adding glutamate to the culture medium of HT-22 cells (Fig. 3A*i*–*iii*), and then washed and incubated the rounded-up ferroptotic dying cells with fresh medium containing GSH. Time-lapse live-cell microscopy of the cultures demonstrated that the same rounded-up dying cells, washed and incubated with GSH-containing culture medium, regained a normal morphology (Fig. 3A*iv*–*vi*) and then proliferated (Movie 4). Parallel cultures of ferroptotic cells that were washed and then maintained in medium with no GSH did not reverse ferroptosis but proceeded to plasma membrane rupture and death (Fig. 3B*i*,*ii*). Over 97% of GSH-treated cells can regain normal morphology and survive (Fig. 3B*ii*,C, Fig. S1B), revealing that this is a general phenomenon in the cell population. The PrestoBlue assay was used as above to demonstrate the restored intracellular reducing environment of the GSH-treated recovered cells (Fig. 3D). These results indicate that GSH can promote reversal of glutamate-induced ferroptosis in the HT-22 cells.

### Glutathione and Ferrostatin-1 promote reversal of erastin-induced ferroptosis

**Fig. 4.**
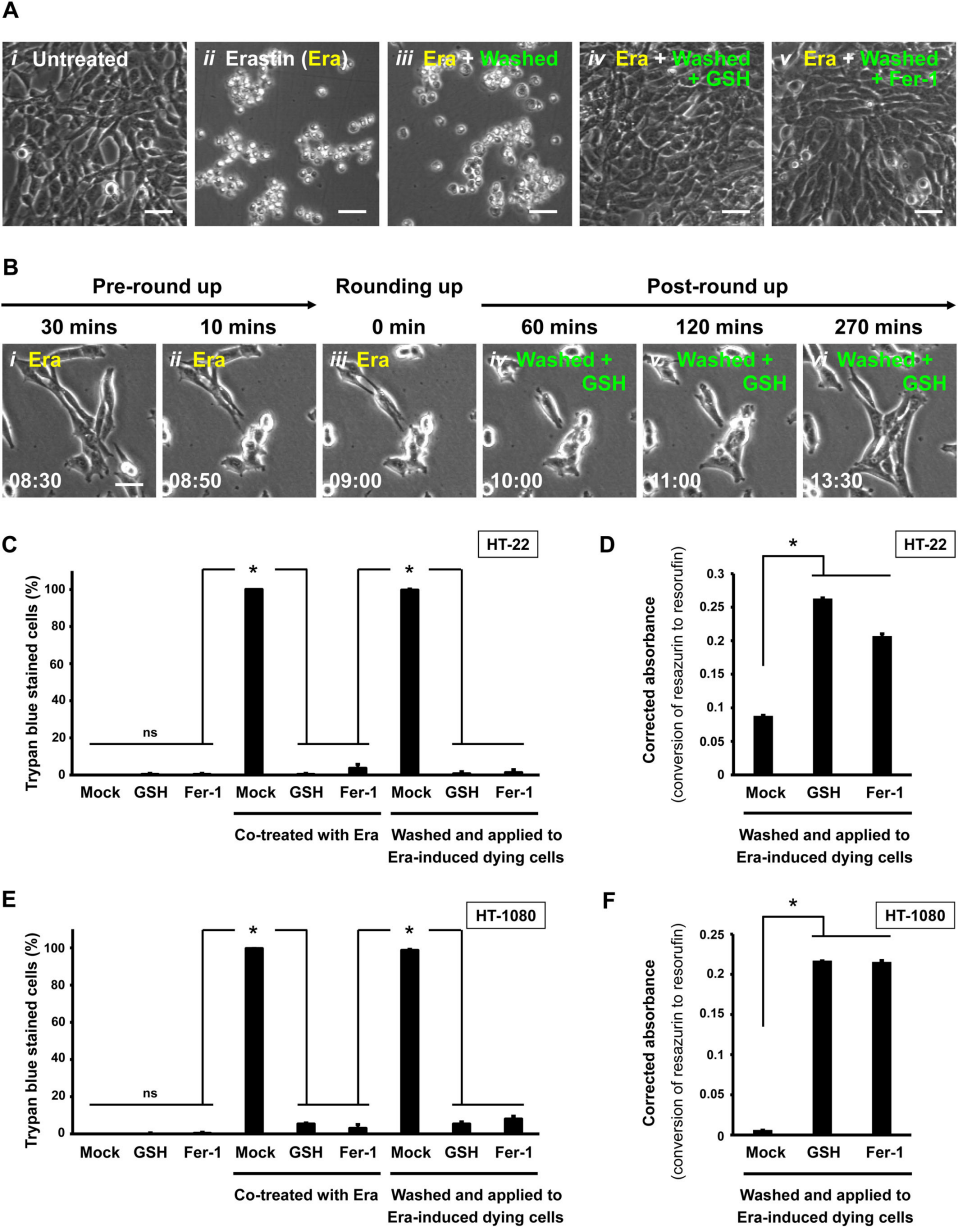
**Reversal of ferroptosis by GSH or Fer-1 in erastin-induced HT-22 and HT-1080 dying cells.** (A) Phase contrast images of HT-22 cells 24 h after the following treatments. Nothing added to the culture medium (*i*), erastin (Era, 10 µM) added to the medium (*ii*), 10 µM Era in the medium for 9 h (cell rounding evident) followed by washing and incubation in medium with no additives (*iii*), like *iii*, but with 1.2 mM GSH added to the wash and incubation medium (*iv*), or like *iii*, but with 10 µM Fer-1 added to the wash and incubation medium (*v*). Scale bars: 60 μm. (B) Time-lapse live-cell phase contrast microscopy of HT-22 cells showing reversal of Era-induced ferroptosis promoted by GSH. Cells were induced with 10 µM Era and imaged 30 min and 10 min before showing signs of rounding (*i*,*ii*), at the time rounding was first noticed (*iii*), and 60, 120 and 270 min after washing to remove Era and continuing incubation in medium containing 1.2 mM GSH (*iv*–*vi*). Same group of cells shown over time (h:min). Scale bar: 20 μm. (C) Percentage of HT-22 cells that displayed plasma membrane permeability and scored as dead in the Trypan Blue vital dye exclusion assay after the following treatments. The first set of cultures was incubated with no additives (Mock) or with 1.2 mM GSH or 10 µM Fer-1 in the culture medium. The second set was like those in the first set, but the culture medium also contained 10 µM Era. The third set was induced with 10 µM Era for 9 h (cell rounding evident) and then washed and incubated in medium with nothing added (Mock) or with 1.2 mM GSH or 10 µM Fer-1 added. Dead cells were counted as those showing plasma membrane rupture with full permeability to the vital stain Trypan Blue. (D) Corrected absorbance for the reduction of resazurin to resorufin in HT-22 cells induced with 10 µM Era for 9 h to initiate ferroptosis, and then washed and incubated for 24 h with fresh medium containing no additives (Mock) or containing 1.2 mM GSH or 10 µM Fer-1. (E) Percentage of HT-1080 cells showing plasma membrane permeability and scored as dead in the Trypan Blue vital dye exclusion assay after the following treatments. The first set of cultures was incubated with no additives (Mock) or with 1.2 mM GSH or 10 µM Fer-1 in the culture medium. The second set was like those in the first set, but the culture medium also contained 10 µM Era. The third set was induced with 10 µM Era for 7.5 h (cell rounding evident) and then washed and incubated in medium with nothing added (Mock) or with 1.2 mM GSH or 10 µM Fer-1 added. Dead cells were counted as those showing plasma membrane rupture with full permeability to the vital stain Trypan Blue. (F) Corrected absorbance for the reduction of resazurin to resorufin in HT-1080 cells induced with 10 µM erastin for 7.5 h to initiate ferroptosis, and then washed and incubated for 24 h with fresh medium with nothing added (Mock) or with 1.2 mM GSH or 10 µM Fer-1 added. Data presented as means±s.d. of three independent experiments. Student's *t*-test: *P<0.001; ns, not significant.

Can GSH and Fer-1 promote cell recovery from ferroptosis that is triggered by a different ferroptosis inducer and in a different cell line? It has been demonstrated that the small molecule erastin triggers robust ferroptosis in HT-22 and human fibrosarcoma HT-1080 cell lines (Dixon et al., 2012; Xie et al., 2016). For this experiment, we first tested the reversibility of ferroptosis in erastin-induced HT-22 after the dying cells had been washed and incubated with fresh culture medium only, or with medium containing either GSH or Fer-1. With no induction of cell death, healthy HT-22 cells divided and spread on the substrate (Fig. 4A*i*). When ferroptosis was induced by erastin, the cells first rounded up (Fig. 4B*i–iii*) and then display plasma membrane rupture (Fig. 4A*ii*,C), as previously reported (Dixon et al., 2012; Xie et al., 2016). In agreement with our findings with glutamate-induced HT-22 cells, we observed that after washing and incubating erastin-induced rounded-up ferroptotic cells with fresh medium, ferroptosis was not reversed and the dying cells progressed to death (Fig. 4A*iii*,C). However, as observed when glutamate was used to induce ferroptosis in HT-22 cells, adding GSH or Fer-1 to the wash and recovery medium did promote reversal of erastin-induced ferroptosis, as indicated by recovery to a normal cell morphology (Fig. 4A*iv*,*v*,B*iv*–*vi*,C, Movie 5) and intracellular reducing environment (Fig. 4D), and also cell division (Movie 5).

**Fig. 5.**
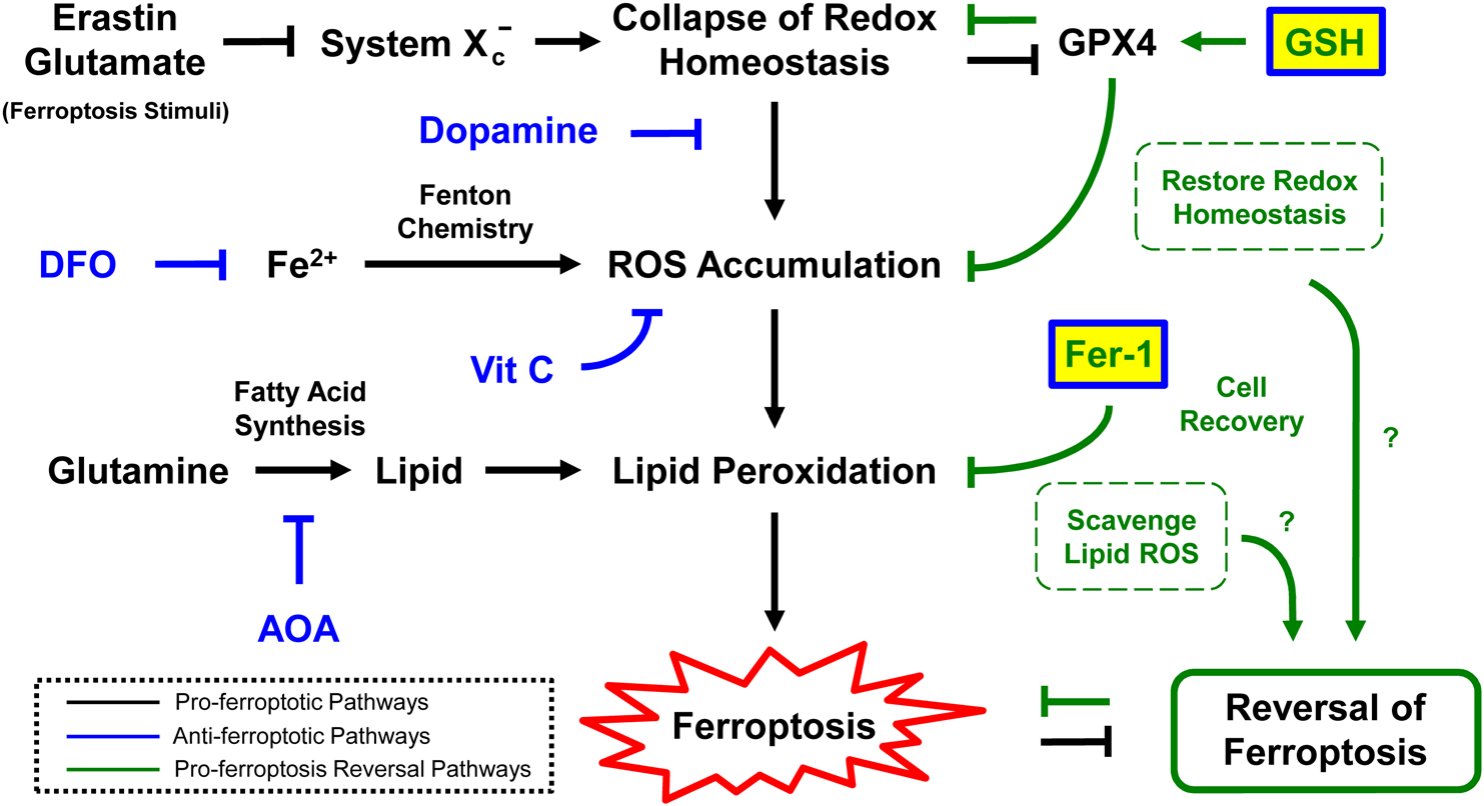
**Proposed model for reversal of ferroptosis.** Interactions between pro-ferroptosis reversal pathways and pro-ferroptosis pathways during reversal of ferroptosis.

Similar results were observed when erastin-induced ferroptosis was tested in HT-1080 cells. When ferroptosis was induced in HT-1080 cells by erastin, they continued progressing to cell death when washed and maintained in normal culture medium (Fig. 4E). However, when the erastin was washed away and cultures were grown with GSH or Fer-1 added to the medium, ferroptosis was reversed in over 90% of the cells (Fig. 4E). Besides, intracellular reducing environment of rescued cells were restored (Fig. 4F). These results in HT-1080 cells are in agreement with our findings for the reversal of glutamate- (Figs 2 and 3) and erastin-induced ferroptosis in HT-22 cells (Fig. 4A–D). In summary, our results document the efficacy of GSH and Fer-1 in reversing ferroptosis (Fig. 5) in both neuronal (HT-22) and fibrosarcoma (HT-1080) cells.

## DISCUSSION

When anastasis was coined at 2012, reversal of apoptosis was the only example (Tang et al., 2009, 2012; Tang and Tang, 2018). Here, we discovered a new cell recovery phenomenon by reversing ferroptosis, thereby adding a new example of anastasis. Ferroptosis is a newly identified process of programmed cell death that has been associated with human diseases such as cancers, brain injury, neurodegeneration, heart failure and liver damage (Dixon et al., 2012; Xie et al., 2016; Stockwell et al., 2017; Gao and Jiang, 2018). While the regulatory mechanism and consequences of ferroptosis are just beginning to emerge (Feng and Stockwell, 2018), it may like apoptosis that provides new strategies for increasing the efficiency of cell death during cancer therapy, or minimizing cell death to preserve vulnerable cells such as neurons, cardiomyocytes and hepatocytes during acute tissue injury. Therefore, our finding that ferroptosis is reversible provides new insight and direction towards potential therapeutic strategies for controlling cell death and survival by mediating its reversibility.

While the ferroptotic and apoptotic cell death processes can be reversible, these cell recovery phenomena could be mediated by different mechanisms yet to be identified. Firstly, ferroptosis and apoptosis have their own distinct cell death execution pathways, so that the corresponding dying cells could require different mechanisms to arrest the cell death executioners and to repair the differently caused damages for cell recovery. As examples, during apoptosis, cellular demolition is executed by proteases such as caspases that cleave hundreds of structural and functional proteins, and also DNases such as endonuclease G and DNA fragmentation factors that destroy genomes (Jacobson et al., 1997; Riedl and Shi, 2004; Taylor et al., 2008). After removal of apoptotic stimuli, the recovering cells express heat shock proteins and XIAP that can arrest caspase activation, ICAD/DFF45 to inhibit DNases, and DNA-repair enzyme poly(ADP)-ribose polymerase-1 (PARP) to repair the genome (Tang et al., 2017; Sun et al., 2017; Tang and Tang, 2018). In contrast, ferroptosis is a non-apoptotic form of cell death that is featured by accumulation of ROS that exceeds the capability of the cells to maintain redox homeostasis, leading to lipid peroxidation and subsequently cell death (Yang and Stockwell, 2016). Application of GSH or Fer-1 can promote reversal of ferroptosis, possibly because GSH enhances the GPX4 activity to arrest ROS accumulation (Ursini et al., 1985), while Fer-1 is a ROS scavenger that can remove the excessive cytosolic and lipid ROS (Skouta et al., 2014), thereby restoring the redox homeostasis to allow cell recovery.

While GSH and Fer-1 are known ferroptosis inhibitors, not every ferroptosis inhibitor can promote reversal of ferroptosis. For example, ferroptotic inhibitors such as DFO, AOA, dopamine and vitamin C can only suppress initiation of ferroptosis, but cannot promote the recovery of dying cells after ferroptosis has been initiated. It could be because these inhibitors are targeting the upstream pathways for initiating ferroptosis, rather than acting on the downstream pathways for executing cell death. For example, DFO is an iron chelator that depletes iron and prevents the iron-dependent accumulation of lipid ROS (Dixon et al., 2012). AOA is a small molecule transaminase inhibitor that blocks the metabolism of glutamine to alpha-ketoglutarate for fatty acid synthesis (Wise et al., 2008). Dopamine is a neurotransmitter that blocks GPX4 degradation (Wang et al., 2016). Vitamin C is an antioxidant to scavenge free radicals in the aqueous phase (Englard and Seifter, 1986; Levine et al., 1986). All of these four inhibitors act on the upstream pathways of lipid peroxidation, while GSH and Fer-1 act on the associated mechanisms for removing lipid ROS as mentioned above. These also suggest potentially distinct regulations between preventing and reversing ferroptosis, yet to be identified. Taken together, our findings that GSH and Fer-1 reverse ferroptosis suggest regulators associated with GPX4 activity and lipid peroxidation as candidate mediators of ferroptosis reversibility.

We and other groups demonstrated that removal of apoptotic stimuli is sufficient to allow reversal of apoptosis to occur *in vitro* and *in vivo* (Tang et al., 2009, 2012, 2015a,b, 2017; Wang et al., 2014; Ding et al., 2016; Sun et al., 2017; Tang and Tang, 2018). In contrast, our present *in vitro* study shows that removal of ferroptosis stimuli such as erastin from HT-1080 cells, and also erastin or glutamate from the HT-22 cells is not sufficient to enable these ferroptosis-initiated cells to recover. It seems that washing and incubating with fresh culture medium alone cannot rescue ferroptotic cells. However, we cannot exclude the possibility that some types of dying cells with robust capability to restore the redox balance could recover without the supplement of GSH or Fer-1, yet to be discovered.

Our discovery that ferroptosis is reversible leads to fundamental questions that remain to be answered. For example, can reversal of ferroptosis occur in live animals? It is technically challenging to track ferroptosis *in vivo*, as the cells recovered from ferroptosis are morphologically indistinguishable from healthy non-ferroptotic cells. While cells that reverse ferroptosis or apoptosis need to repair different kinds of cellular damage, might they share a master regulator or signal that triggers the initiation of anastasis? Further, what are the long-range physiological, pathological and therapeutic implications of this cell recovery process? Answers to these questions and identification of molecular regulators of the pathways involved are under investigation. Elucidating the molecular mechanism and agents that mediate ferroptosis and its reversal will provide us the knowledge and tools needed to explore, understand and exploit the cell recovery process of anastasis.

## MATERIALS AND METHODS

### Materials

All reagents were obtained from MilliporeSigma (Burlington, MA, USA), unless stated otherwise.

### Cell culture

The immortalized mouse hippocampal neuronal HT-22 cell line was obtained from Dr Richard Sang Un Lee (Johns Hopkins University School of Medicine, USA). The human fibrosarcoma HT-1080 cell line was purchased from the American Type Culture Collection. Cells were cultured in Dulbecco's Modified Eagle's Medium supplemented with 10% fetal bovine serum, 100 U/ml penicillin and 100 μg/ml streptomycin (Gibco, Gaithersburg, USA) at 37°C in an atmosphere of 5% CO_2_/95% air. Cells were plated onto Corning tissue culture dishes (35 mm, Corning, USA) with seeding density 3.6×10^5^, and were cultured for 1 day to reach 60% confluency, before being subjected to experiment.

### Induction, inhibition, and reversal of ferroptosis

Ferroptosis in HT-22 cells was induced by 10 mM of glutamate (Glu) or 10 µM erastin (Era), and in HT-1080 cells by 10 µM erastin. Ferroptosis inducers Glu and Era were diluted to final experimental conditions concentrations in fresh medium before adding to cells. To inhibit initiation of Glu- or Era-induced ferroptosis, the Glu or Era was first pre-mixed into the fresh cell culture medium with one of the ferroptosis inhibitors such as aminooxyacetic acid (AOA, 2 mM), deferoxamine mesylate salt (DFO, 100 µM), dopamine hydrochloride (Dopa, 5 µM), ferrostatin-1 (Fer-1, 10 µM), L-glutathione reduced (GSH, 1.2 mM), or vitamin C (ascorbic acid, 0.5 mM), before they were applied to the cells. Removal of ferroptotic inducers was accomplished by washing cells three times with culture medium. To test reversal of ferroptosis, rounded-up ferroptotic dying cells were washed with and cultured in the fresh culture medium containing 10 µM Fer-1 or 1.2 mM GSH.

Of note: apart from ferroptosis inhibitors, DFO has been reported as a cell cycle blocker (Farinelli and Greene, 1996), and AOA impacts on amino acid metabolism and fatty acid synthesis (Wise et al., 2008). These could contribute to the phenomena of the cells that had been co-treated with DFO and glutamate (Fig. 1A*iii*) or AOA and glutamate (Fig. 2A*ii*) displaying lower cell density than their corresponding controls, such as the untreated cells (Fig. 1A*i*), as well as the cells co-treated with vitamin C and glutamate (Fig. 1A*iv*), dopamine and glutamate (Fig. 2A*iii*), or Fer-1 and glutamate (Fig. 2A*iv*).

### Confocal microscopy

Cells were cultured on glass-bottom (MatTek Corporation, Ashland, USA) or plastic (Cloning) culture dishes, which were mounted by adapter to the stage of LSM 780 confocal inverted microscope (Carl Zeiss, Jena, Germany) equipped with an environmental control chamber to maintain 37°C and 5% CO_2_. Time-lapse images were captured with a transmitted light detector T-PMT for phase contrast or differential interference contrast (DIC) microscopy, or with a GaAsP detector for fluorescence signals of mitochondria and nuclei with excitation at 561 and 405 nm respectively, using a 10×, NA 0.3 Plan-Neofluar objective, or a 40× NA 1.4 Plan-Apochromat objective. Images were analysed using Zen 2013 or AxioVision 4.2 software (Carl Zeiss).

### Trypan Blue exclusion assay

Cell death with plasma membrane rupture was determined by staining with the dye Trypan Blue. Live cells with intact cell membranes exclude the dye, but dead cells with permeable plasma membranes allow its penetration. Cells were collected by gentle pipetting or trypsinization, resuspended in 0.2% Trypan Blue solution (Gibco), and subjected to cell count such as by hemocytometer using an inverted microscope to determine the percentage of dead cells versus live stain-excluding cells. Cell viability assays were done in triplicate, counting at least 100 cells for each condition. Statistical comparison was performed using two-tailed Student's *t*-test. Differences were considered to be significant when the *P* value was <0.05.

### PrestoBlue cell viability assay

The intracellular reducing environment of cells and viability, were measured by using the PrestoBlue reagent (Invitrogen, Carlsbad, USA), as instructed by the manufacturer. Resazurin, the active ingredient of PrestoBlue reagent, is converted to resorufin in the intracellular reducing environment of live cells. Conversion of resazurin to resorufin changes its colour and absorbance. PrestoBlue diluted 1:10 with culture medium was incubated with the cells *in situ* at 37°C with 5% CO_2_ for 3 h. Absorbance was measured using a SpectraMax M2 microplate reader (Molecular Devices, San Jose, USA) at 570 nm, using 600 nm as a reference wavelength for normalization (Erb and Ehlers, 1950; Prabst et al., 2017). Background fluorescence of medium alone was subtracted from all the values. Triplicates were performed for each condition. Statistical comparison was performed using two-tailed Student's *t*-test. Differences were considered to be significant when the *P* value was <0.05.

## Supplementary Material

Supplementary information
