# Supplementary material for: Cell recovery by reversal of ferroptosis
Source: Biol Open. 2019 Jun 15;8(6):bio043182. doi: 10.1242/bio.043182 (PMC6602333; doi:10.1242/bio.043182)
Supplement: Supplementary information [file biolopen-8-043182-s1.pdf]

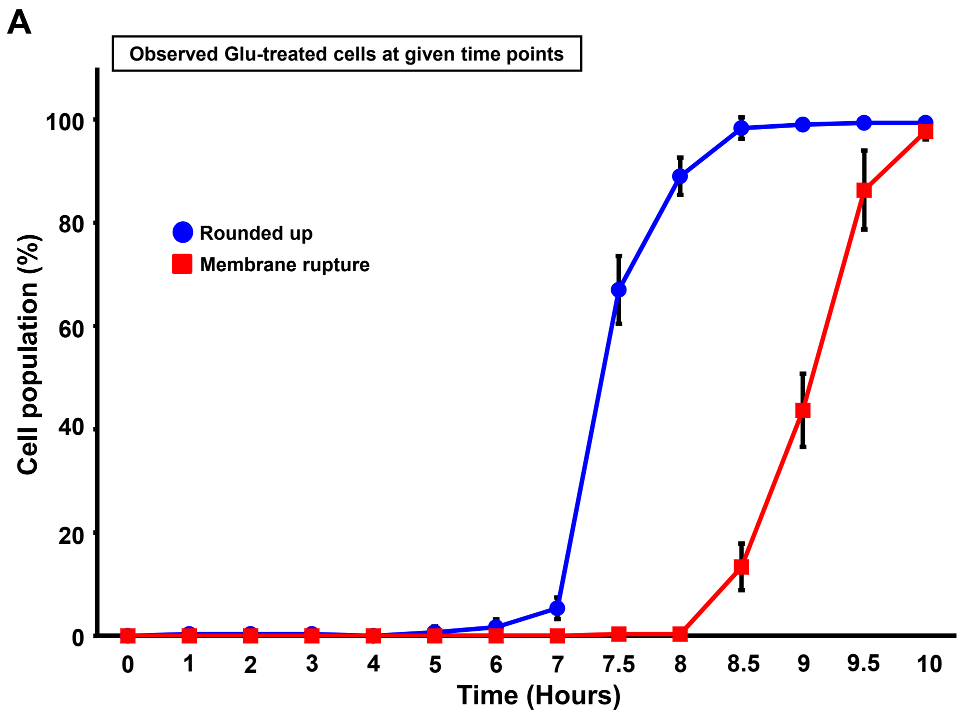
A
Observed Glu-treated cells at given time points
Rounded up
Membrane rupture
Cell population (%)
0
20
40
60
80
100
0 1 2 3 4 5 6 7 7.5 8 8.5 9 9.5 10
Time (Hours)

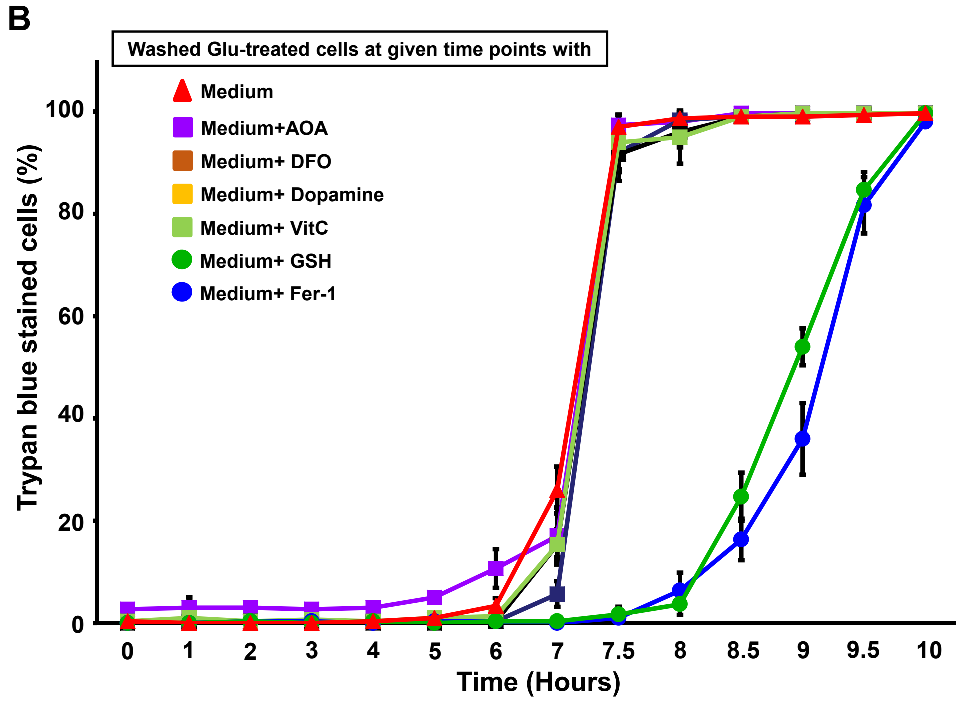
B
Washed Glu-treated cells at given time points with
Medium
Medium+AOA
Medium+ DFO
Medium+ Dopamine
Medium+ VitC
Medium+ GSH
Medium+ Fer-1
Trypan blue stained cells (%)
0
20
40
60
80
100
0 1 2 3 4 5 6 7 7.5 8 8.5 9 9.5 10
Time (Hours)

Fig. S1. Rescue of glutamate-induced ferroptotic dying HT-22 cells with ferroptosis inhibitors at different stages of cell death induction.

A) Percentage of HT-22 cells displayed rounded-up morphology and plasma membrane rupture in response to 10mM glutamate induction (Glu) at 0, 1, 2, 3, 4, 5, 6, 7, 7.5, 8, 8.5, 9. 9.5, and 10 hours.

B) Percentage of HT-22 cells that displayed plasma membrane permeability in Trypan blue exclusion assay, after the following treatments. The cells were first treated with 10mM glutamate (Glu) for 0, 1, 2, 3, 4, 5, 6, 7, 7.5, 8, 8.5, 9. 9.5, and 10 hours. At each time points, the corresponding groups of cells were washed with fresh cell culture medium, and then incubated with or without one of the ferroptosis inhibitors for 24 hours, including aminooxyacetic acid (AOA, 2mM), deferoxamine mesylate salt (DFO, 100µM), dopamine hydrochloride (Dopamine, 5µM), vitamin C (VitC, 0.5mM), ferrostatin-1 (Fer-1, 10µM), or L-glutathione reduced (GSH, 1.2mM).

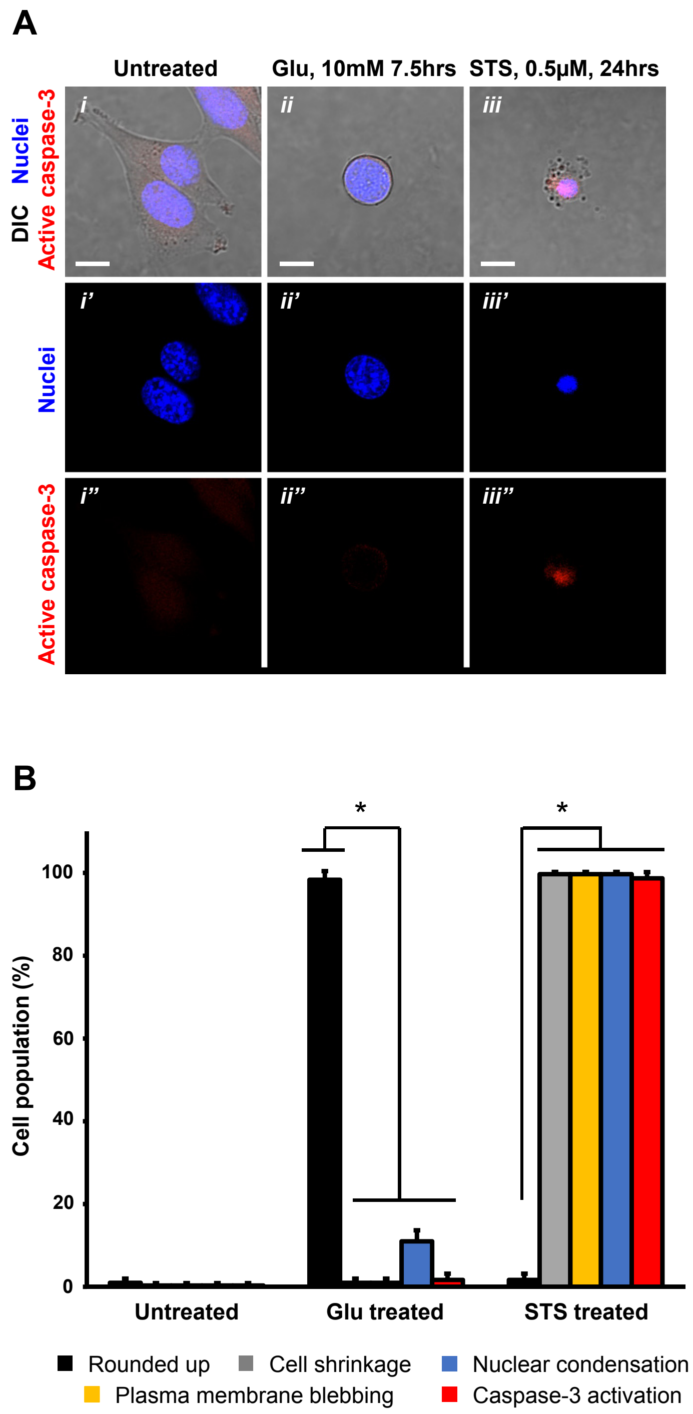
A
Untreated
Glu, 10mM 7.5hrs
STS, 0.5µM, 24hrs
DIC
Nuclei
Active caspase-3
i
ii
iii
i'
ii'
iii'
i''
ii''
iii''
B
Cell population (%)
100
80
60
40
20
0
Untreated
Glu treated
STS treated
Rounded up
Cell shrinkage
Nuclear condensation
Plasma membrane blebbing
Caspase-3 activation

Fig. S2. Hallmarks of ferroptotic and apoptotic HT-22 cells.

A) Confocal microscopy of untreated HT-22 cells (panels ***i***), the cells treated with ferroptosis inducer 10mM glutamate (Glu) for 7.5 hours (panels ***ii***), and the cells treated with apoptosis inducer 0.5µM staurosporine (STS) for 24 hours (panels ***iii***). Cells were stained with Hoechst 33342 (blue fluorescence, nuclei), and then treated the cells with or without cell death inducer as mentioned above, in the presence of NucView 530 caspase-3 substrate (labelling caspase-3 activated cells with red fluorescence) in the culture medium. Merged images of nuclei (blue fluorescence), the caspase-3 substrate for active caspase-3 (red fluorescence) and DIC for cell morphology (top row). Images of nuclei only (middle row). Images of active caspase-3 only (bottom row). Scale (10µm) is shown by bar at lower left of panel ***i***.

B) Percentage of untreated, Glu- or STS-treated HT-22 cells in culture that displayed rounded-up morphology (black), cell shrinkage (grey), plasma membrane blebbing (brown), nuclear condensation (blue), and caspase-3 activation. Data presented as means ± SD of three independent experiments. Student's *t* test: *$P < 0.001$.

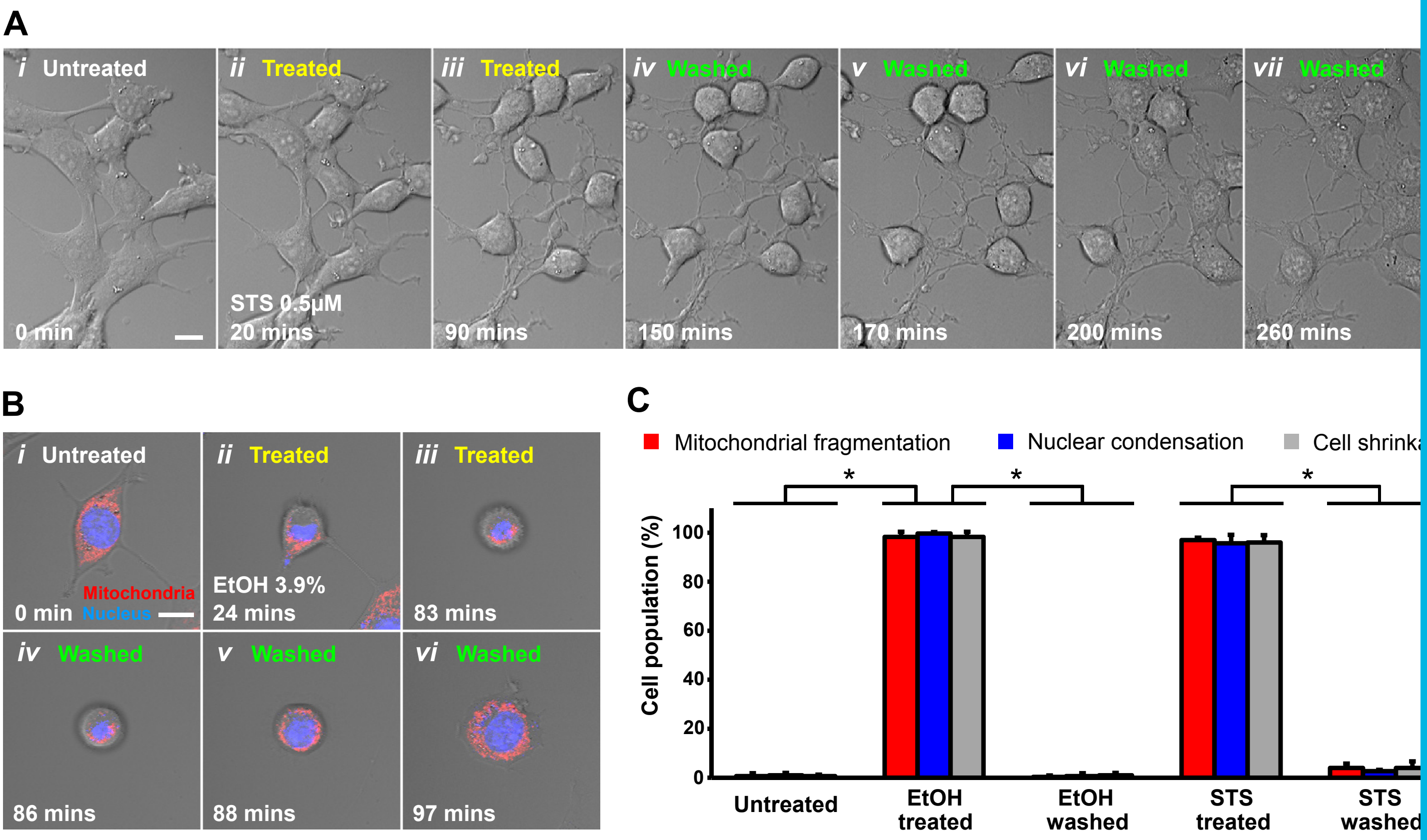

Fig. S3. Reversibility of apoptosis in HT-22 cells.

A) Time-lapse live-cell DIC microscopy of the same HT-22 cells before, during, and after exposure to staurosporine (STS). The same group of cells before staurosporine induction (panel ***i***), 20 and 90 minutes after inducing with 0.5µM staurosporine in cell culture medium (panels ***ii*** and ***iii***), and then after washing and further culturing for 150, 170, 200, and 260 minutes in fresh medium (panels ***iv–vii***). Time (hr:min) is at lower left. Scale (10µm) is shown by bar at lower right of panel ***i***.

B) Time-lapse live-cell confocal microscopy of single HT-22 cell before, during, and after exposure to ethanol (EtOH). Shown here is the same cell before ethanol induction (panel ***i***), after inducing for 24 and 83 minutes with 3.9% ethanol in cell culture medium (panels ***ii*** and ***iii***), and then after washing and culturing with no EtOH for an additional 3, 5, and 14 minutes (86, 88, and 97 minutes from time 0, respectively, panels ***iv–vi***). Merged images, mitochondria (red fluorescence, stained with Mitotracker Red CMXRos) and nuclei (blue fluorescence, stained with Hoechst 33342) were visualized by confocal, and cell morphology by DIC microscopy. Time (hr:min) is at lower left. Scale (10µm) is shown by bar at lower right of panel ***i***.

C) Percentage of untreated, EtOH- or STS-treated, and EtOH-removed or STS-washed HT-22 cells in culture that displayed mitochondrial fragmentation (red), nuclear condensation (blue), and cell shrinkage (grey). Data presented as means ± SD of three independent experiments. Student's t test: *$P < 0.001$.

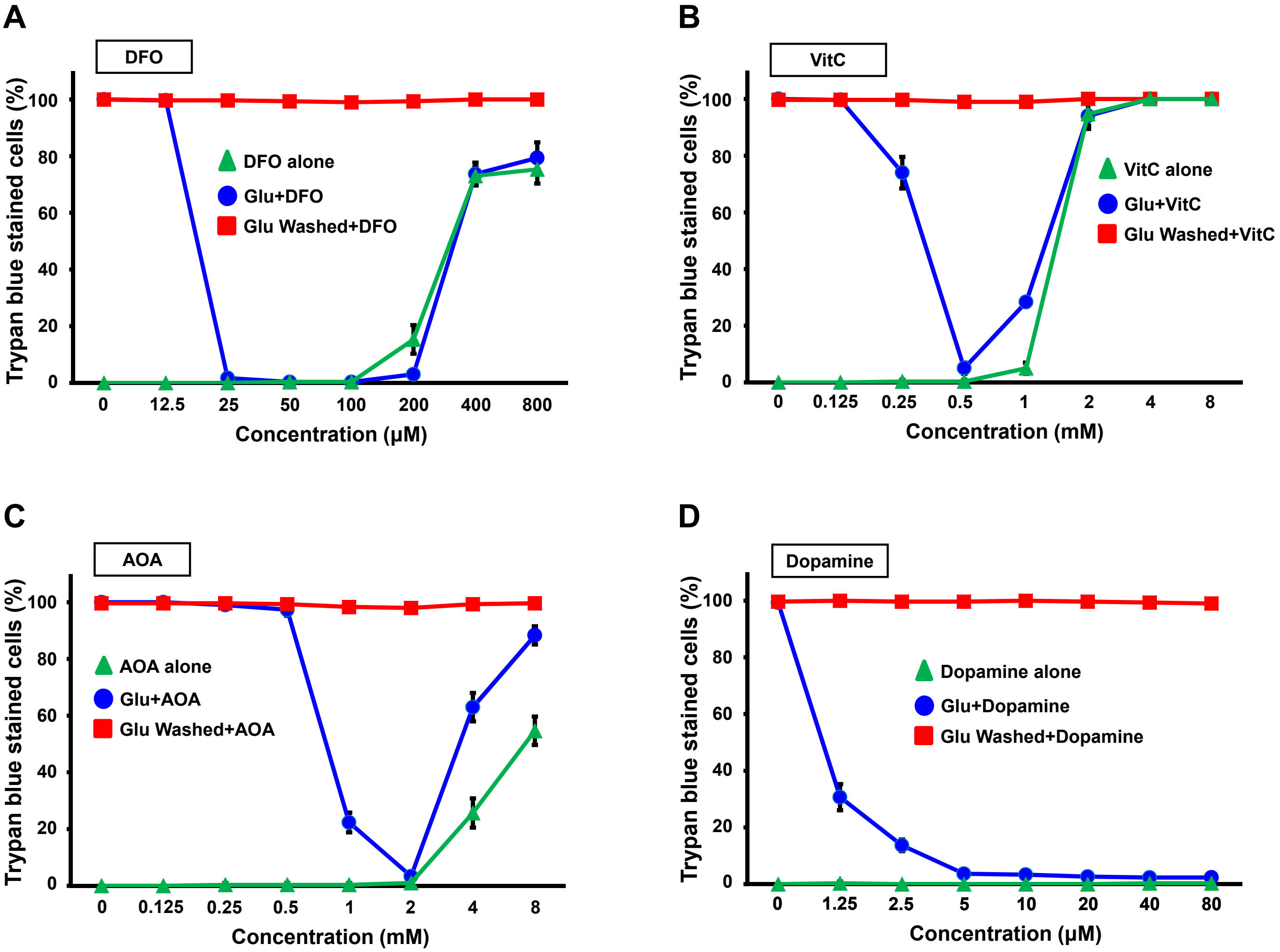

Fig. S4. Dosage responses of glutamate-induced ferroptotic dying HT-22 cells to deferoxamine, aminooxyacetic acid, dopamine and vitamin C.

Percentage of HT-22 cells that displayed plasma membrane permeability in Trypan blue exclusion assay (dead cells) in response to various concentrations of the ferroptosis inhibitors **A)** deferoxamine (DFO), **B)** aminooxyacetic acid (AOA), **C)** dopamine, and **D)** vitamin C (VitC), after being treated as follows. The first set of three cultures were the untreated HT-22 cells incubated in medium containing the corresponding inhibitor with the indicated concentrations for 10 hours (green). The second set of cultures were like the first set, but also contained 10mM glutamate (Glu) in the culture medium (blue). The third set of cultures were the HT-22 cells had first treated with 10mM glutamate for 7.5 hours to initiate ferroptosis, then then washed with the cell culture medium containing the corresponding inhibitor with indicated concentrations for 10 hours (red). Data presented as means ± SD of three independent experiments.

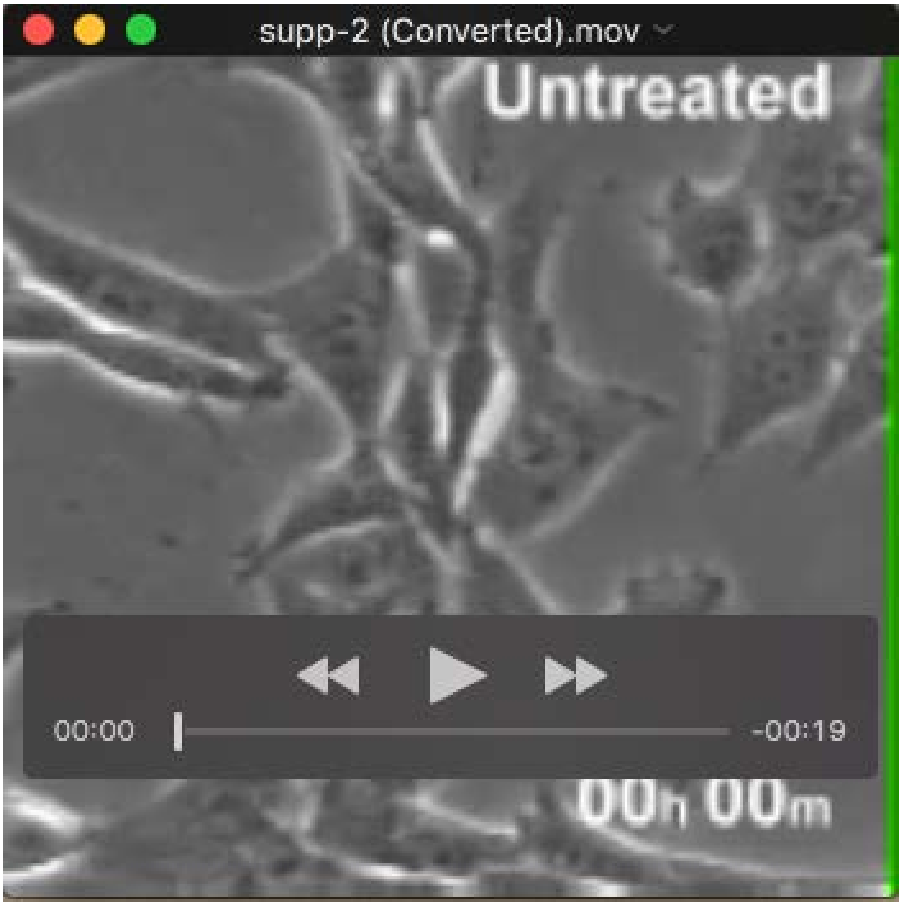

Movie 1. Glutamate-induced ferroptosis in HT-22 cells.
Time-lapse living cell phase contrast microscopy of HT-22 cells before and during glutamate induction. The same HT-22 cells before glutamate induction (Untreated), and induced by 10mM glutamate in culture medium (Glu). Time presented as hr:min.

Biology Open • Supplementary information

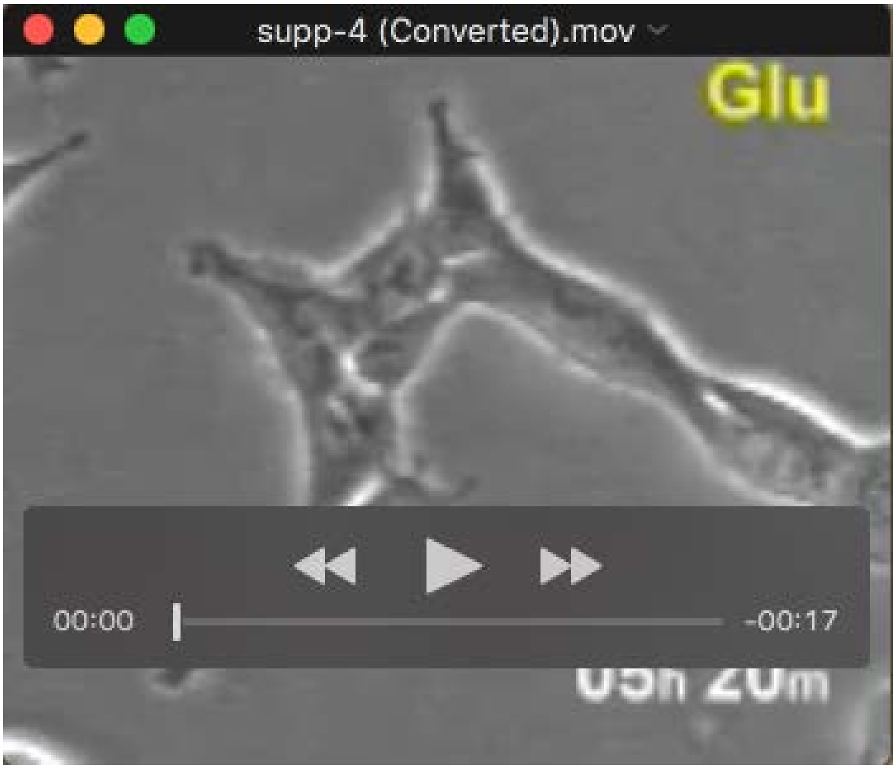

Movie 2. Removal of glutamate from ferroptotic dying HT-22 cells.
Time-lapse living cell phase contrast microscopy of HT-22 cells during and after exposure to glutamate. The same HT-22 cells induced by 10mM glutamate in culture medium (Glu), and then washed with and further cultured in fresh medium (Washed). Time presented as hr:min.

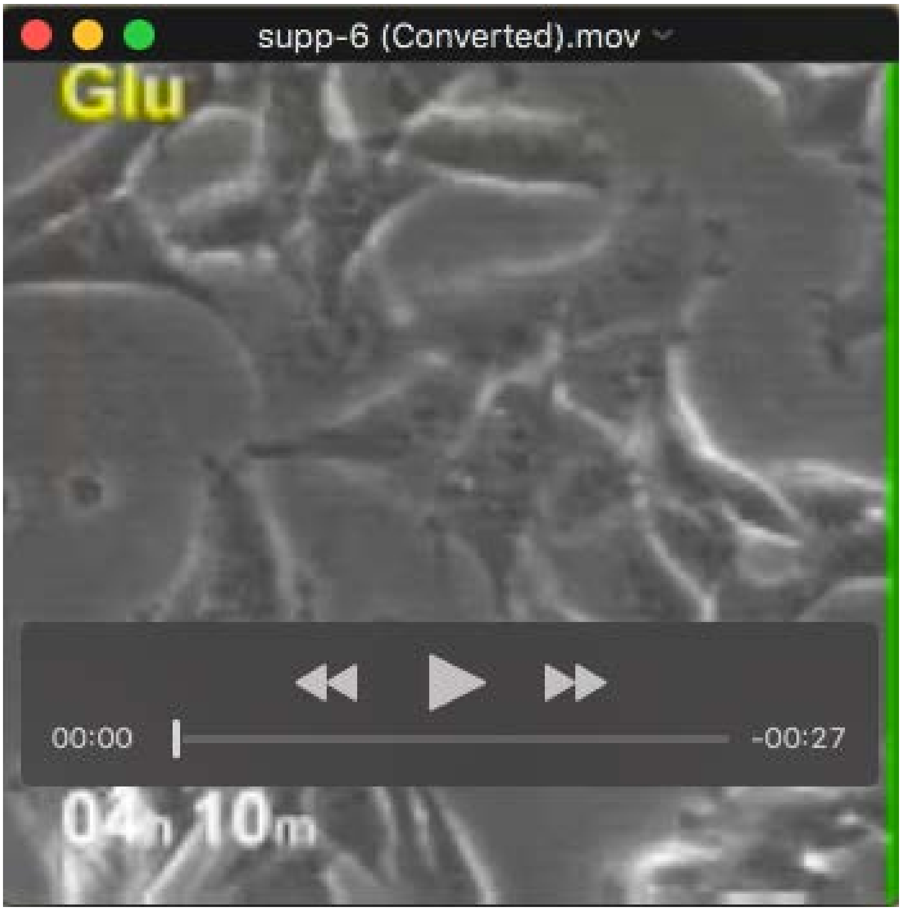

Movie 3. Reversal of glutamate-induced ferroptosis in HT-22 cells with Fer-1 supplement.
Time-lapse living cell phase contrast microscopy of HT-22 cells during exposure to 10mM glutamate (Glu), and then washed with and further cultured in fresh medium containing 10µM ferrostatin-1 (Washed + Fer-1). Time presented as hr:min.

Biology Open • Supplementary information

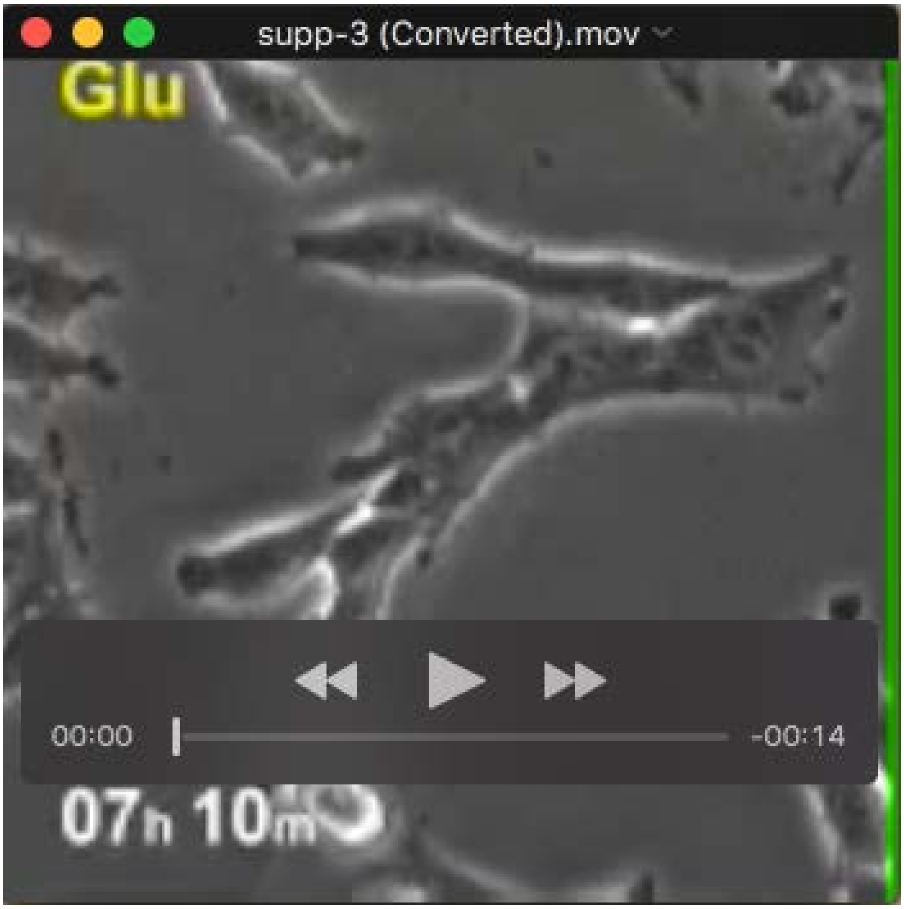

Movie 4. Reversal of glutamate-induced ferroptosis in HT-22 cells with GSH supplement.
Time-lapse living cell phase contrast microscopy of HT-22 cells during exposure to 10mM glutamate (Glu), and then washed with and further cultured in fresh medium containing 1.2mM GSH (Washed + GSH). Time presented as hr:min.

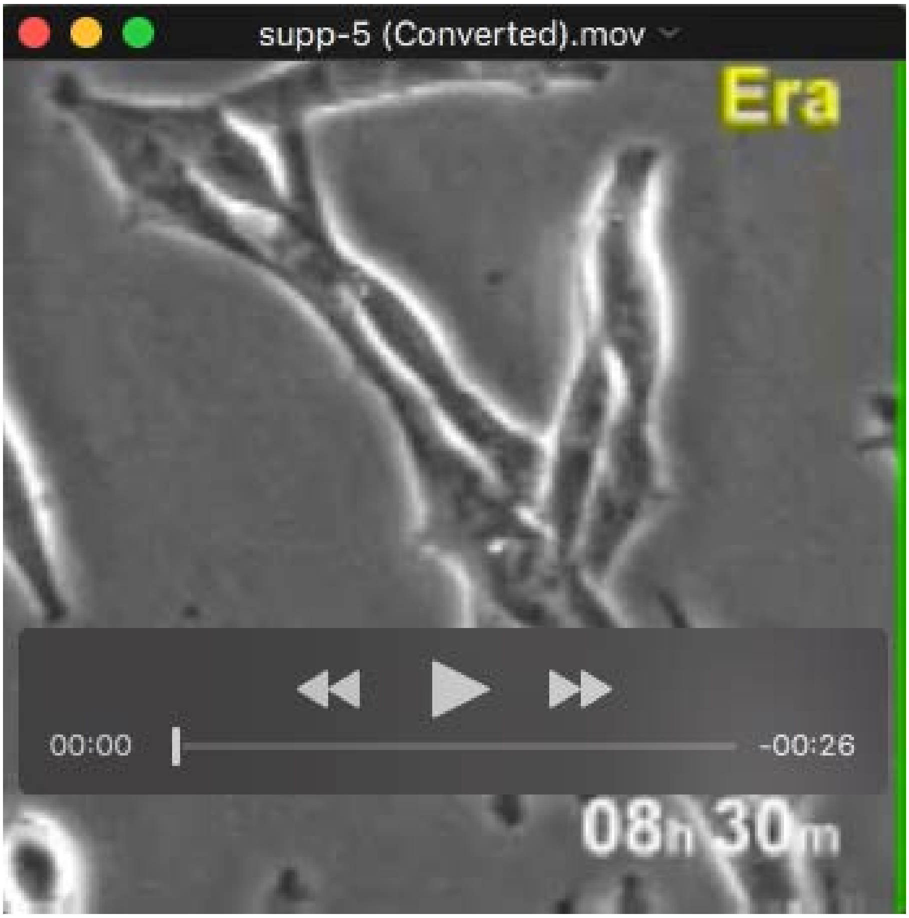

Movie 5. Reversal of erastin-induced ferroptosis in HT-22 cells with GSH supplement.
Time-lapse living cell phase contrast microscopy of HT-22 cells during exposure to 10µM erastin (Era), and then washed with and further cultured in fresh medium containing 1.2mM GSH (Washed + GSH). Time presented as hr:min.
